# National plans and awareness campaigns as priorities for achieving global brain health

**DOI:** 10.1016/S2214-109X(23)00598-3

**Published:** 2024-03-12

**Authors:** Sebastian F Winter, Donna Walsh, Coriene Catsman-Berrevoets, Valery Feigin, Frédéric Destrebecq, Suzanne L Dickson, Matilde Leonardi, Volker Hoemberg, Cristina Tassorelli, Maria Teresa Ferretti, Anna Dé, Antonella Santuccione Chadha, Chris Lynch, Sophia Bakhtadze, Deanna Saylor, Soonmyung Hwang, Kevin Rostasy, Benzi M Kluger, Claire Wright, Phyllis C Zee, David W Dodick, Joke Jaarsma, Mayowa O Owolabi, Jelka Zaletel, Tit Albreht, Rajinder K Dhamija, Anne Helme, Joanna Laurson-Doube, Action Amos, Florence K Baingana, Gus A Baker, Francesca Sofia, Orla Galvin, Tadeusz Hawrot

**Affiliations:** a OneNeurology Partnership, Brussels, Belgium; b International Bureau for Epilepsy, Washington, DC, USA; c Department of Neurology, Massachusetts General Hospital, Harvard Medical School, Boston, MA, USA; d European Paediatric Neurology Society, Paris, France; e Erasmus MC Sophia Children's Hospital, Rotterdam, Netherlands; f World Stroke Organization, Geneva, Switzerland; g National Institute for Stroke and Applied Neurosciences, Auckland University of Technology, Auckland, New Zealand; h European Brain Council, Brussels, Belgium; i Department of Physiology/Endocrinology, Institute of Neuroscience and Physiology, The Sahlgrenska Academy at the University of Gothenburg, Gothenburg, Sweden; j World Federation for Neurorehabilitation, North Shields, UK; k Fondazione IRCCS Istituto Neurologico CarloBesta, Milan, Italy; l International Headache Society, London, UK; m Department of Brain and Behavioral Sciences, University of Pavia, Pavia, Italy; n IRCCS Mondino Foundation, Pavia, Italy; o Center for Alzheimer Research, Karolinska Institute, Stockholm, Sweden; p Women's Brain Project, Bottighofen, Switzerland; q Alzheimer's Disease International, London, UK; r Department of Paediatric Neurology, Tbilisi State Medical University, Tbilisi, Georgia; s World Neurology Foundation, New York, NY, USA; t Department of Neurology, Johns Hopkins University School of Medicine, Baltimore, MD, USA; u Department of Internal Medicine, University Teaching Hospital, Lusaka, Zambia; v Department of Paediatric Neurology, Children's Hospital Datteln, University of Witten/Herdecke, Witten, Germany; w International Neuropalliative Care Society, Roseville, MN, USA; x Department of Neurology, University of Rochester, Rochester, NY, USA; y Meningitis Research Foundation, Bristol, UK; z Confederation of Meningitis Organisations, Bristol, UK; aa World Sleep Society, Rochester, MN, USA; ab Department of Neurology, Center for Circadian and Sleep Medicine, Northwestern University Feinberg School of Medicine, Chicago, IL, USA; ac International Headache Society Global Patient Advocacy Coalition, London, UK; ad Mayo Clinic College of Medicine, Phoenix, AZ, USA; ae Atria Academy of Science and Medicine, New York, NY, USA; af European Federation of Neurological Associations, Brussels, Belgium; ag Center for Genomic and Precision Medicine, and Neurology Unit, Department of Medicine, College of Medicine, University of Ibadan, Ibadan, Nigeria; ah African Stroke Organization, Ibadan, Nigeria; ai Lebanese American University of Beirut, Beirut, Lebanon; aj Blossom Specialist Medical Center, Ibadan, Nigeria; ak National Institute of Public Health, Ljubljana, Slovenia; al Institute of Human Behaviour and Allied Sciences, New Delhi, India; am Multiple Sclerosis International Federation, London, UK; an International Bureau for Epilepsy African Region, Blantyre, Malawi; ao Regional Advisor, Mental Health and Substance Abuse, World Health Organization African Region, Brazzaville, Congo; ap American Migraine Foundation, New York, NY, USA; aq American Brain Foundation, Minneapolis, MN, USA

## Abstract

Neurological conditions are the leading cause of death and disability combined. This public health crisis has become a global priority with the introduction of WHO's *Intersectoral Global Action Plan on Epilepsy and Other Neurological Disorders 2022–2031* (IGAP). 18 months after this plan was adopted, global neurology stakeholders, including representatives of the OneNeurology Partnership (a consortium uniting global neurology organisations), take stock and advocate for urgent acceleration of IGAP implementation. Drawing on lessons from relevant global health contexts, this Health Policy identifies two priority IGAP targets to expedite national delivery of the entire 10-year plan: namely, to update national policies and plans, and to create awareness campaigns and advocacy programmes for neurological conditions and brain health. To ensure rapid attainment of the identified priority targets, six strategic drivers are proposed: universal community awareness, integrated neurology approaches, intersectoral governance, regionally coordinated IGAP domestication, lived experience-informed policy making, and neurological mainstreaming (advocating to embed brain health into broader policy agendas). Contextualised with globally emerging IGAP-directed efforts and key considerations for intersectoral policy design, this novel framework provides actionable recommendations for policy makers and IGAP implementation partners. Timely, synergistic pursuit of the six drivers might aid WHO member states in cultivating public awareness and policy structures required for successful intersectoral roll-out of IGAP by 2031, paving the way towards brain health for all.

## Introduction

Neurological conditions are the leading cause of mortality and disability combined, ranking first in disability-adjusted life-years (DALYs) and second as a cause of global deaths: neurological conditions account for 9 million deaths per year.^1^ At least one in three people will develop a neurological condition in their lifetime, at a cost exceeding US $1·7 trillion in Europe and the USA alone.^2^ More than half of all countries have an increasing risk of death from neurological conditions, making these the fastest-growing cause of death among non-communicable diseases (NCDs).^3^ By 2040, neurological conditions are projected to increase DALYs by approximately 50%.^1^ This public health crisis is driven by global population growth, ageing societies, improved treatment options for once-lethal conditions, food insecurity, armed conflict, lifestyle changes, post-COVID-19 conditions,^4^ environmental pollution, and climate change.^1^, ^5^ Although neurological conditions affect many individuals of all ages worldwide, most of the burden (78·5% deaths and 77·3% DALYs) is in low-income and middle-income countries (LMICs).^5^ Compared with high-income countries, LMICs are more likely to face substantial resource constraints (eg, insufficient access to essential medicines, medical infrastructure, and medical equipment); disproportionate neurological workforce shortages (eg, on average, there are three adult neurologists per 10 million people in LMICs *vs* 475 adult neurologists per 10 million people in high-income countries; a >150-fold difference);^6^ negative health determinants (eg, low public awareness and health literacy levels, poverty, widespread stigma, or discriminatory legislation); and catastrophic out-of-pocket health expenditures.^7^ These widening global neurological health disparities underscore the urgency for capacity building and prioritised national action plans across LMICs.^7^

In response to this growing public health crisis, WHO launched the Intersectoral Global Action Plan on Epilepsy and Other Neurological Disorders 2022–2031 (IGAP)^8^ in May, 2022, with 194 member states committing to “reduce the stigma, impact and burden of neurological disorders…and improve the quality of life of people with neurological disorders, their carers and families”.^8^ Additionally, a WHO brain health position paper^9^ was published as an IGAP technical complement, defining brain health as “the state of brain functioning across cognitive, sensory, social-emotional, behavioural and motor domains, allowing a person to realise their full potential over the life course, irrespective of the presence or absence of disorders”.^9^ These milestones, which mark a pivotal shift in global health policy and a neurology revolution, mandate WHO and member states to act decisively in addressing brain health and the escalation of neurological burden.^10^, ^11^

## Two IGAP priority targets for accelerated rollout of the 10-year plan

IGAP contains five strategic objectives and ten global targets for member states to achieve by 2031.^8^ Governments are tasked with domestication and intersectoral rollout of the IGAP framework to deliver innovative, contextualised, and integrated programmes that surpass conventional public health measures. Domestication involves setting context-specific national targets that address local priority needs and challenges, paired with clear indicators for evaluating progress. IGAP-directed policies should be intersectoral, reflecting the interconnectedness of neurological and brain health, and wider societal, politico-legislative, economic, and environmental contexts.^8^ A participatory and human rights-based approach to neurology will ensure that outcomes advance the rights of people with neurological conditions.

A successful national IGAP response thus requires unprecedented levels of intersectoral governance and collaboration. Continuous technical support (eg, strategic guidance, educational resources, and policy co-design) to governments by WHO and the global neurology community is crucial. Appropriate learnings can be drawn from the national dementia plans developed under the WHO *Global Dementia Action Plan 2017–2025*.^12^ Here, early engagement and alignment of national and local implementation partners on priority actions proved vital to plan development and motivation to deployment.^12^, ^13^

The WHO Brain Health Unit is co-creating an IGAP implementation toolkit with neurology stakeholders to provide practical resources and recommendations for policy makers. Additionally, a WHO-led global neurology status report featuring country-level baseline data on IGAP targets will monitor nations’ progress and foster accountability. To map health infrastructure, the World Federation of Neurology—a key implementation partner—is developing a global needs registry and core curriculum for neurology.^14^ Governmental use of these forthcoming IGAP instruments is paramount, but their use will be contingent on sufficient collective awareness, political will, and prioritisation of neurological and brain health in national agendas.

The OneNeurology Partnership is a global multistakeholder consortium uniting international neurological organisations to stimulate collaborative advocacy, action, and accountability for prevention, treatment, and management of neurological conditions worldwide. In this Health Policy, OneNeurology Partnership representatives and allied global neurology stakeholders call for WHO member states and neurology stakeholders to prioritise attention towards two IGAP targets, to expedite national delivery of the entire 10-year plan. The two priority targets correspond to IGAP's first strategic objective, to *“*raise policy prioritisation and strengthen governance”^8^ and contain the following country-level deliverables: first, “75% of countries will have adapted or updated existing national policies, strategies, plans or frameworks to include neurological disorders by 2031.”^8^ Second, “100% of countries will have at least one functioning awareness campaign or advocacy programme for neurological disorders by 2031.”^8^

Successful public health agenda rollouts require effective national action plans and clear governance frameworks, as evidenced in areas such as HIV and AIDS,^15^ antimicrobial resistance,^16^ cancer,^17^ and dementia.^13^ Only a dedicated national plan or programme ensures a policy commitment that will be robust enough for enactment and financing of the planned activities. Similarly, national advocacy and awareness campaigns can yield measurable improvements in awareness and health behaviours.^18^, ^19^, ^20^, ^21^, ^22^ Widespread awareness fosters bottom-up advocacy, which is a principal civic lever to translate public health needs into political action. Simultaneously, public awareness of issues enhances support for top-down policies (eg, awareness of links between alcohol and cancer increases public support for alcohol regulatory policies).^23^ Collective attainment of the identified priority targets can thus synergistically expedite the entire IGAP agenda.

However, 18 months after IGAP adoption, progress on these two deliverables remains insufficient. National neurological and brain health plans (NBHPs) and campaigns are urgently needed in LMICs, less than 30% of which have any existing policies dedicated to neurological conditions.^6^ Lessons from the public health response to dementia illustrate that most countries struggle with prompt national action plan formulation. For example, of the 194 member states committing to a dementia plan in 2017, just 39 countries achieved this crucial goal by May, 2023. To meet WHO's 2025 target of 146 plans, 54 new plans are required annually (75% of member states).^13^ In response, Alzheimer's Disease International launched the #WhatsYourPlan campaign, galvanising governments to formulate, finance, and implement dementia plans.^13^ Since campaign inception in 2021, 20 additional WHO member states pledged to create national plans, underscoring the power of campaigning to accelerate policy prioritisation. Prompt, concerted action towards priority targets is thus essential for countries to meet their 2031 IGAP commitments.

To this end, we propose six strategic drivers for adoption by member states, including policy makers, civil society organisations, and cross-sectoral stakeholders: universal community awareness, integrated neurology approaches, intersectoral governance, regionally coordinated domestication, lived experience-informed policy, and neurology mainstreaming (figure).

**Figure:**
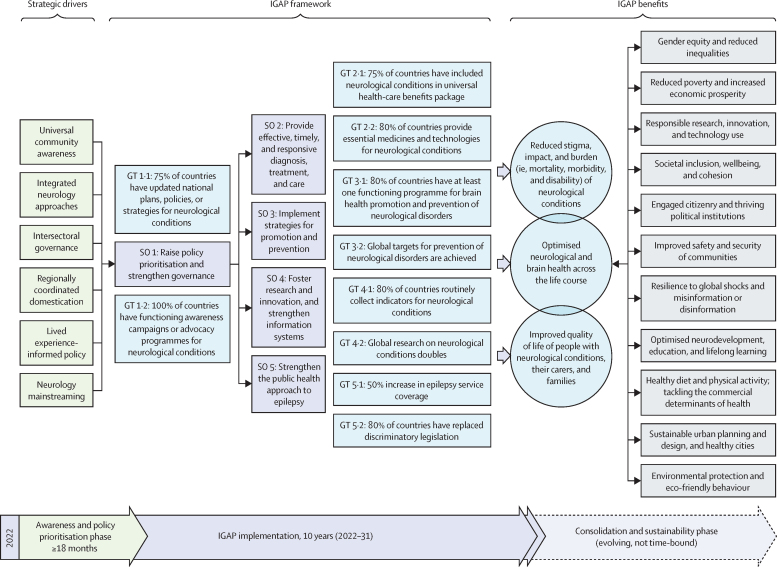
Proposed framework to accelerate IGAP implementation, and to promote global neurological and brain health Depicted are the six strategic drivers for timely achievement of the IGAP SO 1, and associated global targets GT 1·1 and 1·2, which we identify as the priority targets to accelerate rollout of the entire IGAP plan. The overarching 10-year IGAP outcomes are contextualised with the WHO brain health strategy (blue circles), alongside the wider societal determinants and the benefits of optimised neurological and brain health (grey rectangles). GT=global target. IGAP=*Intersectoral Global Action Plan on Epilepsy and Other Neurological Disorders 2022–2031*. SO=strategic objective.

The following analysis introduces a pragmatic framework to expedite national IGAP delivery by leveraging these six drivers over the next 18 months and beyond. Drawing on insights from relevant global health agendas and emerging IGAP initiatives, it provides actionable recommendations and essential resources for policy makers and implementers. Moreover, it explores the challenges and opportunities in IGAP domestication and intersectoral policy design, emphasising the diverse societal benefits of fostering neurological and brain health for all.

## Six strategic drivers to achieve IGAP priority targets

### Universal community awareness: translating IGAP and promoting brain health literacy, contextualised with lived experience

Widespread community awareness of IGAP and brain health directives is fundamental to policy prioritisation. At a minimum, this requires translation of IGAP, a technical high-level policy framework, into content with tangible actions that are reflective of lived experience. The relevance, core messages, testimonials, and implications and recommendations that are accessible to policy makers, neurology stakeholders, and the general public must be conveyed. As lived experience varies with sociocultural, life course, geographical, and sex-related and gender-related differences, tailored translational efforts are crucial. Emerging examples include WHO's *Intention to Action Series: People Power* report^24^ and person-centred educational materials and campaigns by civil society organisations (appendix p 1). Resource dissemination is effective when linked to community interventions. A notable example is the international movement of memory cafes,^25^ which provide peer support, social connection, and cognitive stimulation for people with dementia and their carers, enabling cost-effective access to education, information, support, and services.

Effective measures need systematic scaling, given low public awareness around IGAP and brain health, particularly in LMICs.^8^, ^14^ Beyond fostering grassroots advocacy and policy prioritisation, universal community awareness brings essential public health gains. Cultivating a collective understanding of the brain and the entire nervous system, as our most vital lifelong asset, is fundamental to prevention in neurology.^9^ Stroke, which leads in neurological DALYs and disproportionately affects people in LMICs, is largely preventable;^26^ yet, evidence-based pragmatic strategies remain severely underused.^27^ Brain health literacy programmes emphasising key determinants, preventive actions, and benefits of optimised brain health necessitate integration into primary health-care and community services, especially in rural or remote areas.^28^

Appropriate awareness and engagement of individuals living with neurological conditions, their carers, and their families (ie, co-production of care) effectively enhances the entire neurological care trajectory (ie, prevention, early detection, treatment, management, rehabilitation, and palliative support). Awareness measures should provide quality information on cardinal risk factors, treatment options, and appropriate support structures.^29^ Comprehensive interventions enhance public recognition of neurological signs and symptoms (including sex-specific and gender-specific differences), helping to improve understanding, respect, and support for affected individuals, and promoting active participation in neurological care and rehabilitation.

Currently, 92% of individuals with neurological conditions report experiencing stigma caused by knowledge gaps, misconceptions, and misinformation, and discriminatory legislation surrounding these conditions and their frequently invisible nature.^8^, ^11^, ^30^ Similarly, people with visible impairments or mobility aids face societal participation barriers, including few disability-inclusive accessible spaces in most LMICs. Effects on quality of life, psychosocial wellbeing, societal participation, educational and professional milestone attainment, health care seeking, and treatment adherence can be deleterious. Awareness measures must target multilevel stigma and educate about human rights protections, including the UN *Convention on the Rights of Persons with Disabilities*,^31^ ratified by 186 member states.

The final measure in need of systematic scaling is the promotion of advocacy for access to medicines (IGAP global target 2.2).^8^ Despite the advent of transformative disease-modifying and gene-modifying drugs, their exorbitant costs limit availability, especially in LMICs. For example, onasemnogene abeparvovec—a gene therapy for spinal muscular atrophy—is US Food and Drug Administration approved but costs $2 million per dose.^32^ Civil society organisations can markedly influence access and affordability issues, as exemplified by the Multiple Sclerosis International Federation's application for the inclusion of multiple sclerosis treatments in the WHO Essential Medicines List (EML). Three multiple sclerosis disease-modifying therapies were subsequently added to the EML, setting precedent for improving medicines access in LMICs and offering a potential blueprint for future EML applications.^33^ Concurrently, enhancing access to cost-effective medicines already listed on the EML, such as epilepsy and Parkinson's disease medications, remains vital. Advocacy should promote national medicines policies, regulatory and procurement frameworks (covering quality assurance, pooled procurement, innovative financing, and capacity building), and rational drug selection and use.^8^

### Integrated neurology approaches: leveraging strategic entry points and building multistakeholder partnerships (six Ps coalition)

Traditional so-called silo mentality in health care and disease-specific advocacy might cause effort fragmentation, insufficient resource allocation, missed collaboration opportunities, division of public attention, confusion of policy makers, and commonalities among neurological conditions to be overlooked.^11^ Instead, national NBHPs necessitate holistic, biopsychosocial, and integrated approaches. Investments should prioritise strategies targeting shared systemic challenges and solutions across disease categories, whether in neurology advocacy or service delivery. Integrated programmes are probably most impactful in LMICs, offering a cost-effective, pragmatic, and sustainable approach to the massive neurological burden. With more than 400 neurological conditions (and related disease-specific advocacy groups), individuals’ needs are more similar than different, spanning prevention (eg, shared risk factors and health behaviours), correct diagnosis, appropriate treatment, rehabilitation, and palliative care. Access to education, employment, social inclusion and reintegration, and active participation constitute further shared priorities. Integrated approaches are both achievable and effective, as evidenced in fields such as cancer, which has more than 200 distinct types differing in manifestation and treatment response.^34^

Strategic entry points can catalyse integrated neurology approaches in both policy advocacy and service delivery. Countries might broaden existing, well functioning disease-specific initiatives as entry points for building comprehensive neurological infrastructures. WHO has highlighted epilepsy as one such entry point given its shared public health challenges (ie, gaps in treatment, research, prevention, and inclusion) and association with other neurological conditions (stroke, neurotropic communicable diseases, neurodegenerative conditions, perinatal brain injuries, traumatic brain injuries, brain neoplasms, genetically determined neurocognitive impairments, developmental encephalopathies, comorbid psychiatric conditions, sleep–wake disorders, etc).^8^, ^35^

Multistakeholder partnerships underpin and sustain integrated approaches: a unified neurology community can propel governmental NBHP adoption through concerted advocacy. Additionally, civil sector public–private partnerships can plug crucial service provision gaps when national plans are unattainable or disjointed (as experienced in the dementia response).^13^ In 2023, Owolabi and colleagues^11^ proposed an expanded neurology stakeholder ecosystem to enhance global synergistic actions on brain health, known as the six Ps coalition: patients, health-care service and product providers, policy makers, payors, implementation partners, and the general population.^11^ The overarching term brain health^9^ is creating new political momentum and unity among diverse neurology stakeholders.^36^ Still, the opportunities, risks, and questions that this new terminology brings need addressing. For example, grouping peripheral nervous system conditions or mental health^37^ with brain health remains challenging and controversial. Historically viewed as distinct, linking these terms requires intensive collaboration across the six Ps to ensure consensus, coherent messaging, and inclusivity.

Finally, integrated approaches can yield gains beyond neurology by enhancing surveillance, prevention, treatment, and rehabilitation for multiple neurological conditions and NCDs at once. For instance, in Norway, from 1990 to 2019, a coordinated approach addressing the triple threat of stroke, dementia, and ischaemic heart disease significantly reduced age-standardised incidence rates of dementia by 5·4%, ischaemic heart disease by 30·0%, and stroke by 35·3%. These reductions were achieved by monitoring shared risk factors, implementing preventive interventions, and enhancing care services.^38^ Drawing from WHO's NCD Best Buys,^39^ which target primary NCD drivers (ie, tobacco, unhealthy diets, alcohol, and physical inactivity) at a cost-effectiveness ratio of 100 international dollars or less per DALY averted in LMICs, countries might consider adapting a Brain Best Buys approach, given overlapping risk factors.^40^

### Intersectoral governance: fostering neurological Health in All Policies

Any successful action plan, including NBHPs, hinges on several pivotal factors. These factors are sufficient political leadership and commitment, key stakeholder involvement (including lived experience representatives), context-specific strategies targeting unmet needs and gaps, well defined targets retaining adaptability, progress tracking against baselines, sufficient resource allocation, and accountability mechanisms reinforced by robust monitoring and evaluation frameworks.^41^

Beyond these universal considerations, a distinct driver of effective NBHPs is intersectionality. Neurological and brain health reciprocally interacts with diverse domains beyond health and research, including economic,^42^ social,^43^, ^44^ educational,^45^ political,^40^, ^46^, ^47^ and environmental^48^, ^49^ sectors (table; appendix pp 1–3). This multidimensionality necessitates consolidation into NBHPs. Similarly, quality of life is vital for individuals, families, and carers but enhanced only through integrated services extending beyond health care. Moreover, the chronicity of most neurological conditions requires longitudinal strategies supporting active societal involvement. Effective NBHPs will thus address individual and population health needs along with broader neurological health determinants across the life course.

**Table:** Multidimensionality of neurological and brain health beyond the health sector: implications for intersectoral policies, integrated governance, and brain health-directed policy making

	**Neurological and brain health interdependence**	**Policy implications and recommendations**
Economics, labour, and financial security	Global costs of neurological conditions exceed trillions per year (US$);^50^, ^51^, ^52^ healthy and safe workplaces, and financial security are major brain health determinants;^9^, ^53^ brain health in ageing populations has major economic impacts;^42^ poverty–disability cycle: work absence, carer burden, and out-of-pocket payments impoverish many households in LMICs^51^, ^52^	Prioritise cost-effective interventions (eg, stroke prevention strategies^54^ and WHO Best Buys^39^); taxation (eg, of tobacco, salt, alcohol, and sugar) funds health policies, and tackles neurological and non-communicable disease risk factors;^26^ shift to a longevity economy by promoting healthy ageing;^55^ foster virtuous cycles: reinvest economic gains into public health thus reducing poverty and enhancing (brain) health; implement financial and social protection schemes (eg, health insurance, disability pension, tax benefits, and work protection)^9^
Politics and societal cohesion	Population health impacts democracy and societal cohesion:^56^ brain health might foster a more resilient, engaged, and prosocial population;^40^, ^46^, ^47^ unconscious neurophysiological processes influence political attitudes, identities, and behaviours^57^	Holistic societal concepts (mental and brain capital)^58^, ^59^ might yield quantifiable brain health metrics as policy benchmarks; launch prosocial-directed community interventions^60^ (eg, WHO *Commission on Social Connection*); apply political neuroscience to policy making to reduce emotional fallacies, cognitive bias, polarisation, and misinformation; enhance decision making and policy forecasting^57^
Development and education	Many countries lack systems to monitor crucial, early brain development (first 1000 days);^61^, ^62^ more than 250 million children in LMICs might not reach their full developmental potential;^63^ prenatal and postnatal drug exposure (including to alcohol and nicotine) has neurotoxic effects, which is aggravated by societal biases;^64^ scant prevention measures for youth in LMICs elevate risks for avertable conditions (eg, perinatal brain injuries, and meningitis)^5^ and delay neurological treatment; early education and lifelong learning fosters resilience, cognitive reserve, and health behaviours (eg, reducing stroke and dementia risks)^65^	Implement WHO's *Global Scales for Early Development*^62^ for improved child development tracking and resource allocation; align NBHPs with evidence-informed drug policies, awareness campaigns, and educational programmes; NBHPs must prioritise early prevention strategies: perinatal care, vaccinations, and prenatal and neonatal neurometabolic screening; align NBHPs with national educational policies;^9^ launch brain health literacy programmes for parents and educators to enhance and safeguard neurodevelopment
Sex and gender equity	Sex and gender influence both prevalence and burden of neurological conditions;^43^, ^66^ women's under-representation in clinical research worsens health service inequities;^43^, ^66^ informal caregiver burden increases women's economic risks,^67^ pension gaps,^68^ and old-age poverty thus deepening gender inequities^43^	NBHPs should incentivise equitable neurological research and care: balanced clinical trials and specialised training on sex and gender factors in neurology;^43^ provide effective carer support: training, financial aid, pensions, preventive health care; fund scalable carer education (eg, WHO iSupport)^28^
Infrastructure	Infrastructure design impacts societal participation and healthy (brain) behaviours; few LMICs have disability-inclusive infrastructure, limiting access and inclusion; improving road safety is crucial in LMICs; accident-related TBI and spinal injury are prevalent^69^	NBHPs should guide infrastructure policies, informed by emerging frameworks^70^ and neurourbanism concepts;^71^ redesign both educational and workplace settings: incentivise safe, smoke-free, inclusive, neurodiverse, and adaptable settings; NBHPs should reinforce member states' road safety commitments (eg, WHO *Global Plan for the Decade of Action for Road Safety*)
Environment and climate change	Pollution and climate change decrease neurological^49^, ^72^ and mental health;^73^ LMICs face disproportionate effects due to limited environmental, infrastructural, and health and safety regulations^72^	Harmonise NBHPs with environmental policies given anthropogenic effects on the neural exposome;^48^ boost access to green and blue spaces (ie, areas of surface water) to enhance (brain) health;^74^ brain health promotion might foster ecofriendly behaviours via enhanced metacognitive abilities^75^
Food and agriculture	Adequate nutrition (ie, healthy, balanced diets) promotes lifelong brain health;^9^ adverse effects on brain health include agricultural (pesticide use), sanitary (foodborne neurotropic infections), and industrial factors (ultra-processed foods)	Tackle commercial health determinants via marketing restrictions, consumer education, and product taxation, reformulation, and labelling;^76^ NBHPs can inform public-facing nutrition policies (eg, promoting breastfeeding and brain-healthy food programmes)^9^, ^77^
Technology and digitalisation	The AI–neurotechnology nexus harbours vast potential for neurology and brain health (eg, brain-to-text decoding^78^); digitalisation affects brain health, including neurodevelopment, cognition, and social behaviours^79^	Develop policies for safe, effective, and ethical AI use in neurology, informed by WHO's AI for health regulatory considerations;^80^ NBHPs should inform digital literacy policy: AI use in education, workforce upskilling, and adaptation to automation^53^
Ethics and human rights	Dignity neuroscience identifies human rights as emergent properties that are critical for brain health;^81^ address ethicolegal challenges of generative AI (rights to education, work, privacy, data protection, etc)^82^ and neurotechnology (eg, cognitive privacy and liberty);^83^ discriminatory laws and multilevel stigma against people with neurological conditions (eg, epilepsy) persist in many countries^84^	Leverage global AI^82^ and neurotechnology^85^ guidelines for holistic, evidence-informed policy design; explore dignity neuroscience as a universally applicable ethical concept;^81^ NBHPs should reinforce member states' commitments to the UN *Convention on the Rights of Persons with Disabilities*; use WHO-OHCHR guidelines^86^ to align laws and practices with international human rights obligations

AI=artificial intelligence. LMICs=low-income and middle-income countries. NBHPs=neurological and brain health plans. OHCHR=Office of the United Nations High Commissioner for Human Rights. TBI=traumatic brain injury.

We propose a neurological Health in All Policies approach to IGAP implementation, with NBHPs collaboratively developed, aligned, and executed across key governmental sectors, including health, education, employment, social services, science, and technology (table; appendix pp 1–3). Engaging other relevant ministries, such as culture, public safety, justice, finance, economic development, environment, food, agriculture, urban and rural development, and transportation, can amplify effects. Successful intersectoral governance demands exceptional collaboration and diplomacy, involving civil and private sectors, supported by health ministries. While achieving neurological Health in All Policies nationally is crucial, cascading intersectoral governance models to the community level will maximise impact. WHO's *Toolkit for Developing a Multisectoral Action Plan for Noncommunicable Diseases*^87^ offers transferable insights for national and local policy makers to enhance intersectionality in NBHPs and IGAP-directed policy design.

### Regionally coordinated domestication: harmonisation of global and national IGAP targets

Member states, particularly LMICs with constrained health system capacity and financial resources, should delineate national priority actions that address critical unmet needs and promise substantial return on investment. Focusing on a set of priority targets and prioritising cost-effective and operational interventions (Best Buys) represents a pragmatic, high-yield strategy to curb the growing neurological burden in LMICs. IGAP domestication (ie, effectively transposing IGAP into context-specific national NBHPs) is foundational. Accounting for unique national challenges, critical gaps, infrastructure and resource considerations, and health system characteristics optimises NBHP operability, efficacy, and impact. Similarly, context-specific performance indices across countries and regions must be established.

Regionally coordinated harmonisation of the IGAP framework with existing national efforts can markedly accelerate national NBHP and campaign attainment. Regional consortia, especially WHO regional offices, should serve as knowledge brokers supporting governments with IGAP domestication and intersectoral alignment. Early IGAP regionalisation and domestication activities are emerging across regions, including Africa, Europe, the Americas, and south Asia (appendix pp 4–5). For example, the WHO Regional Office for Africa is pioneering an IGAP situational analysis, outlining priority neurological conditions and high-yield policy recommendations for the continent. Co-creation with key regional and national civil stakeholders shall ensure context-specificity and actionability at country level. Similar neurological policy ecosystems are warranted in other regions, ideally as WHO-supported regional IGAP consortia. Core responsibilities of these consortia would encompass IGAP adaptation based on shared regional priorities, supporting NBHP development, identifying key implementers, coordinating allocation and use of shared resources, facilitating good practice exchange, overseeing monitoring and accountability, and establishing health data-sharing governance among regional member states.

### Lived experience-informed policy making: prioritising meaningful engagement of people with neurological conditions, their carers, and families

Successful NBHP design entails mapping the needs of people with lived experience and devising well resourced policies to address them. Policy making informed by lived experience (ie, person-centred, needs-based co-creation of policies) requires early, meaningful, and well coordinated patient and public involvement (PPI). Lessons from public health responses to cancer (eg, *European Guide for Quality National Cancer Control Programmes*^34^) and diabetes (eg, *Guide for National Diabetes Plans—CHRODIS+*^41^, ^88^) show that early and sustained PPI proved instrumental to national plan development, adoption, and implementation. The new WHO framework for meaningful engagement of people living with NCDs, and mental health and neurological conditions^83^, ^89^ can guide lived experience-informed NBHP design.

Given the absence of strong PPI structures in many countries, decision makers and budget holders should promote neurological patient-caregiver associations and incentivise formation of national neurological alliances. Representing people with neurological conditions, national policies can cover a number of aspects: coordinating potent national awareness campaigns, jointly advocate for NBHPs, monitor progress and accountability; and serve as PPI focal points throughout IGAP implementation. Similarly, global consortia such as the OneNeurology Partnership are well placed to support governments in fostering and fortifying national neurology policy making.

### Neurology mainstreaming: positioning neurological and brain health into broader policy agendas

IGAP underpins the mandate for national NBHPs and awareness campaigns, whereas neurology mainstreaming (ie, advocating to embed neurological and brain health into broader policy agendas) effectively amplifies policy prioritisation. Aligned with IGAP's intersectoral nature, this driver offers flexibility for alternative pathways to governmental prioritisation when NBHPs and neurology-specific campaigns are difficult to achieve. Alternative pathways to prioritisation might include devising or adapting a broader national brain health strategy encompassing both mental and neurological conditions, or integrating neurology into an existing NCD plan containing IGAP-directed targets. Integration into an NCD plan also highlights unmet needs for global alignment of existing WHO NCD policies and programmes with neurology and IGAP. Doing so would reinforce the 2018 UN High Level Political Declaration on NCD prevention and control, which recognised mental and neurological conditions as significant contributors to the global NCD burden.^90^

From an advocacy standpoint, neurology stakeholders must hold governments accountable on other ratified policy frameworks relevant to neurological and brain health (appendix p 5). Consolidating brain health into the UN Sustainable Development Agenda is crucial for reaching the 17 Sustainable Development Goals by 2030, most of which are currently unlikely to be met.^11^, ^91^, ^92^ Effective neurology mainstreaming requires strategic emphasis on IGAP's relevance to core policy issues beyond health, targeting decision makers across sectors. Policy makers must ultimately view IGAP pursuit as vital to, and in alignment with, the success of broader policy agendas, including economic, sociopolitical, and environmental returns (figure; table).

## Conclusions and call to action

IGAP has anchored neurological health as a global societal imperative: all 194 member states have committed to translating this overarching policy framework into an effective national public health response. Yet—nearly 2 years post adoption of the IGAP plan—progress is still inadequate, and attainment of 2031 IGAP targets is increasingly at risk. Lessons from other global health contexts emphasise the importance of national action plans and awareness campaigns to catalyse rollout of public health agendas such as IGAP. To expedite delivery of these priority targets, our suggested framework proposes six strategic drivers with key takeaways for member states.

The first driver is universal community awareness. This driver involves the scale up of person-centred IGAP awareness and brain health literacy programmes for essential public health gains—ie, enhancing prevention, care co-production, and neurology advocacy, thus reducing stigma and discrimination. Second is integrated neurology approaches. This driver involves leveraging strategic entry points (eg, epilepsy) and multistakeholder partnerships (six Ps) for unified neurology advocacy and service delivery targeting shared neurological and systemic challenges. The third is intersectoral governance. The multidimensionality of neurological and brain health can be captured by championing a neurological Health in All Policies approach and emphasising cross-sectoral collaboration in both NBHP design and rollout. Fourth is regionally coordinated domestication. This driver involves harmonising global IGAP targets with national priorities to address critical unmet needs, supported by regional neurological policy ecosystems (eg, WHO-backed regional IGAP consortia). Fifth is lived experience-informed policy. Here, person-centred, needs-based co-creation of policies is ensured through meaningful engagement with people with neurological conditions, and their carers and families from the outset, in coordination with national neurological alliances. Sixth is neurology mainstreaming. This driver involves prominently integrating and highlighting the criticality of neurological and brain health in broader policy agendas, ensuring alternative pathways to policy prioritisation.

In leveraging these drivers effectively, member states can foster an integrated, participatory, and tailored national IGAP response, yielding lasting health, economic, sociopolitical, and environmental benefits, and improving the lives of individuals with neurological conditions, and their carers and families. We call on the global community to strive towards a world where neurological and brain health is valued, enhanced, promoted, and protected across the life course.

## Declaration of interests

DW is Chair (unpaid) and CC-B is Vice-Chair (unpaid) of the OneNeurology Partnership. CT has received grants or contracts from ERA-Net Neuron and AbbVie; consulting fees from AbbVie, Eli Lilly, Teva, Pfizer, Lundbeck, and Dompeé; payment or honoraria for lectures or presentations from AbbVie, Eli Lilly, Teva, Pfizer, and Lundbeck; travel support from AbbVie, Eli Lilly, and Lundbeck; and is past president of the International Headache Society. MTF has received consulting fees from Roche, payment or honoraria for lectures or presentations from Lundbeck, and is the co-founder of Women's Brain Project. KR has received consulting fees from Roche (Operetta trial), and payment or honoraria for lectures or presentations from Merck. BMK has received a grant from the National Institute on Aging; royalties or licences from Elsevier; payment or honoraria for lectures and presentations from the American Academy of Neurology, Parkinson's Foundation, International Parkinson and Movement Disorders Society, and Davis Phinney Foundation; and is President of the International Neuropalliative Care Society. CW is an employee of Meningitis Research Foundation, which has received grants from GlaxoSmithKline, Pfizer, Sanofi Pasteur, Serum Institute, and Tableau Foundation. PCZ has received consulting fees from Idorsia, CVS Caremark, Eisai, and Jazz Pharmaceuticals; has stock or stock options in Teva (spouse); and is President of the World Sleep Society. DWD has received consulting fees from Amgen, Atria, CapiThera, Cerecin, Ceruvia Lifesciences, CoolTech, Ctrl M, Allergan, AbbVie, Biohaven, GlaxoSmithKline, Lundbeck, Eli Lilly, Novartis, Impel, Satsuma, Theranica, WL Gore, Genentech, Nocira, Perfood, Praxis, AYYA Biosciences, Revance, Pfizer, and Perfood; payment or honoraria for lectures or presentations from American Academy of Neurology, Headache Cooperative of the Pacific, Canadian Headache Society, Canadian Pain Society, MF Med Ed Research, Biopharm Communications, CEA Group Holding Company (Clinical Education Alliance), Teva, Amgen, Eli Lilly, Lundbeck, Pfizer, Vector Psychometric Group, Clinical Care Solutions, CME Outfitters, Curry Rockefeller Group, DeepBench, Global Access Meetings, KLJ Associates, Academy for Continued Healthcare Learning, Majallin, Medlogix Communications, Medica Communications, MJH Lifesciences, Miller Medical Communications, WebMD Health/Medscape, Wolters Kluwer, Oxford University Press, and Cambridge University Press; has non-profit board membership with American Brain Foundation, American Migraine Foundation, OneNeurology, International Headache Society Global Patient Advocacy Coalition, Atria Health Collaborative, Arizona Brain Injury Alliance, and Domestic Violence HOPE Foundation/Panfila; has received financial support from the Department of Defense, National Institutes of Health, Henry Jackson Foundation, Sperling Foundation, and Patient Centered Outcomes Research Institute; has stock options in Aural Analytics, ExSano, Palion, Man and Science, Healint, Theranica, Second Opinion/Mobile Health, Epien, Nocira, Ontologics, King-Devick Technologies, EigenLyfe, AYYA Biosciences, Cephalgia Group, and Atria Health; has shares in Axon Theraputics, Ontologics, EigenLyfe, and Cephalgia Group; is on the board of directors for Axon Theraputics, King-Devick Technologies, and Cephalgia Group; and has the following patent (number 17189376.1-1466; Title: Onabotulinum Toxin Dosage Regimen for Chronic Migraine Prophylaxis (non-royalty bearing); patent application submitted: Synaquell (Precon Health). AH is an employee of Multiple Sclerosis International Federation (MSIF); MSIF received funding from BristolMyersSquibb, Sanofi, Merck, Viatris (formerly Mylan), Novartis, Biogen, and Roche over the last 5 years. MSIF's independence and all its donations from the health-care industry are governed by MSIF's health-care policy. MSIF has not received any funding from industry for its access to medicines work in 2019, 2020, 2021, 2022, or 2023. JL-D was an employee at MSIF until October, 2023. OG and TH received institutional support from the European Federation of Neurological Associations in running the OneNeurology Secretariat. All other authors declare no competing interests.
