# Supplementary material for: National plans and awareness campaigns as priorities for achieving global brain health
Source: Lancet Glob Health. 2024 Mar 12;12(4):e697–706. doi: 10.1016/S2214-109X(23)00598-3 (PMC10951964; doi:10.1016/S2214-109X(23)00598-3)
Supplement: Supplementary appendix [file mmc1.pdf]

THE LANCET
Global Health

Supplementary appendix

This appendix formed part of the original submission and has been peer reviewed. We post it as supplied by the authors.

Supplement to: Winter SF, Walsh D, Catsman-Berrevoets C, et al. National plans and awareness campaigns as priorities for achieving global brain health. *Lancet Glob Health* 2024; **12:** e697–706.

Supplementary Appendix

Supplementary Table 1. Emerging examples of IGAP and brain health-related educational resources and awareness measures.

Educational resources (position papers, reports, toolkits, eLearning)	• WHO Brain Health Position Paper[1]
	• WHO Technical Brief Series on Parkinson's Disease[2] and Epilepsy[3]
	• EAN Brain Health Strategy[4]
	• AAN Brain Health Action Plan[5]
	• WHO Intention to action series: people power report[6]
	• IHME Brain Health Atlas
	• EBC Brain Health Policy Roadmap
	• WHO toolkit for dementia-friendly initiatives[7]
	• WHO guidelines on risk reduction of cognitive decline and dementia[8]
	• EFNA IGAP eLearning Series
	• IBE IGAP Unpacked Guide
	• WFN Brain Health Initiative
	• The Brain Health Diplomat's Toolkit (for LAC region)[9]
	• IBE-ILAE-WFN IGAP Webinar Series
	• WFNR 2023 Brain Awareness Week Webinar Series
	• WHO Science in 5: Brain Health Series
Awareness measures (advocacy interventions, campaigns)	• WFN World Brain Day[10]
	• EAN Brain Health Mission
	• IAN Brain Health for All Campaign
	• AAN National Brain Health Day and Brain Health Summit
	• MS Brain Health Initiative
	• MSIF World MS Day
	• CoMO World Meningitis Day
	• IHS migraine and tension-type headache awareness campaigns
Abbreviations: *AAN= American Academy of Neurology; CoMO= Confederation of Meningitis Organisations; EAN=European Academy of Neurology; EBC= European Brain Council; EFNA= European Federation of Neurological Associations; IAN= Indian Academy of Neurology; IBE= International Bureau for Epilepsy; IGAP= Intersectoral Global Action Plan on Epilepsy and Other Neurological Disorders 2022-31; IHME= Institute for Health Metrics and Evaluation; IHS= International Headache Society; LAC= Latin America and the Caribbean; MSIF= Multiple Sclerosis International Federation; WFN= World Federation of Neurology; WFNR= World Federation for Neurorehabilitation; WHO= World Health Organization.*	

Supplementary Table 2. Multidimensionality of neurological/brain health beyond the health sector: implications for intersectoral policies, integrated governance, and brain health-directed policymaking.

Sector/domain	Interdependence with neurological and brain health	Policy implications and recommendations
Economics, labour & financial security	• ***Global economic costs*** attributed to poor mental and neurological health reach trillions of USD annually.[11] Stroke costs approximated USD 900 billion in 2017;[12] dementia's 2019 costs were around USD 1·3 trillion, with informal care impacts accounting for >50% of this expense.[13]<br>• ***European economic costs*** of brain conditions neared EUR 800 billion in 2010, with 40% attributed to indirect expenditure like absenteeism.[14]<br>• ***Work as a social determinant of health***:[15] working conditions, including workplace safety, financial security, social connection, and potential for self-actualisation, significantly impact brain health. [1]	• ***Ensure proportionate resource allocation***: policy cost-benefit analyses must encompass both *direct* healthcare costs and *indirect* costs (i.e., lost neurodevelopmental potential, workforce productivity decline, caregiver burden) of neurological ill-health.<br>• ***Prioritise cost-effective interventions*** (e.g., stroke prevention strategies[16]), drawing from "WHO Best Buys".<br>• ***Taxation of tobacco, salt, alcohol, and sugar*** can generate substantial health-related funding whilst controlling key drivers of major neurological conditions and NCDs.[17]
	• ***The (brain) health of ageing societies*** is associated with profound societal/economic impacts.[18]<br>• ***Individual financial sequelae*** of absenteeism and out-of-pocket expenditures can perpetuate a *poverty-disability cycle* driving many households, especially in LMICs, to medical bankruptcy.<br>• ***Socioeconomic disadvantage*** and financial strain adversely affect brain health; associations include increased dementia risk, and decreased brain volume in children and adults.[1,19]	• ***Shift to a 'longevity economy'***, by promoting healthy ageing, employment in individuals >50 years or older, and education/training at later ages.[20]<br>• ***Reinvest into public health:*** economic growth policies should translate to increased health/social care spending, vital for alleviating poverty and enhancing population-level brain health, thus creating a *virtuous cycle*.<br>• ***Implement financial and social protection schemes*** (i.e., general health insurance, disability pension, tax benefits, employment protection for carers etc.).[1]
Politics & societal cohesion	• ***Global political uncertainties and democratic backsliding:*** global trends indicate a reduced number of democratic nations, the population residing in them, the contraction of democratic rights, and a rise in 'autocratising' countries.[21,22]	• ***Investing into population-level brain health*** can foster a more united, resilient, informed, and engaged citizenry, pivotal for robust political processes and institutions.[24]

	• ***Population health and democratic performance are interrelated***,[23] with neurological/brain health impacting civic engagement, societal cohesion, national security, resilience to global shocks, and susceptibility to mis-/disinformation. [24–26]<br>• ***Unconscious neuro-physiological and emotional processes*** shape political attitudes, identities, and behaviours.[27,28]	• ***Promote social connection and prosociality*** at community-level to address negative (brain) health impacts of loneliness / social isolation and foster health, wellbeing, and longevity (cf. WHO Commission on Social Connection).[29]<br>• ***Whole-of-society concepts*** like "mental capital",[30] mental wealth,[31] and "brain capital"[32] aim to define the collective socio-politico-economic value of brain health. Quantifiable population-level brain health metrics may yield new benchmarks for policymaking.<br>• ***Apply political neuroscience insights to improve policymaking***, i.e., strategies targeting emotional fallacies, cognitive bias, polarisation, mis-/disinformation; enhancing decision behaviours and 'policy forecasting'.[27,28]
Development & education	• ***Early education and lifelong learning*** yield compounding effects, enhancing resilience, *cognitive reserve* (e.g., reducing dementia risk[33]) and promoting primary preventative behaviours (e.g., physical activity and healthy diet to decrease stroke risk).	• ***NBHP should closely integrate with countries' educational policies***, as critical determinants of brain health across the life course.[1]<br>• ***Introduce brain health literacy programmes for parents and educators*** to safeguard/optimize neurodevelopment.
	• ***The first 1000 days***, from conception to age 24 months, are vital for brain development.[34] Globally, over 250 million children <5 years in LMICs are at risk of not reaching their full developmental potential due to poverty, malnutrition, environmental and social hazards.[35,36]	• ***Use WHO's Global Scales for Early Development (GSED)***[37] to standardise monitoring of children's development up to 36 months and allocate resources effectively to improve developmental outcomes.
	• ***Pre- and post-natal drug exposure***, including from alcohol and nicotine, can cause lasting neurotoxic effects.[38] Societal bias on perceived neurodevelopmental impacts of illicit vs. legal substances aggravates this issue.[38]	• ***Align NBHP with updated, evidence-informed drug policies***, awareness campaigns, and educational programs.
	• ***Communicable neurological conditions (particularly meningitis)*** remain the dominant cause of neurological DALYs in children (<5 years), disproportionally affecting LMICs.[39]<br>• ***Underutilisation of preventive measures for young populations*** in many LMICs heightens risks for avertable conditions (i.e., perinatal brain injuries, neurotropic communicable disease), and delays detection/management of non-fatal neurological conditions.	• ***NBHP must prioritise early prevention strategies*:** enhanced perinatal care, vaccinations, and introduction of screening programs (e.g., pre- and neonatal screening for neurometabolic diseases).<br>• ***NBHP should budget for family and carer support structures***, considering the prolonged, substantial (social, economic) impacts associated with caregiving for young individuals.
Sex/gender equity	• ***Sex and gender significantly influence*** prevalence, burden, and progression of neurological conditions.[40,41]<br>• ***Women's underrepresentation in clinical research***, particularly clinical trials, results in inequitable service provision, including delayed diagnosis, inappropriate treatment, and caregiving.[40–42]	• ***NBHP should incentivise equitable neurological research and care provision,*** including sex/gender-balanced clinical trials, sex/gender-disparity awareness measures, and specialised neurological training on sex/gender-specific clinical factors.[40]<br>• ***Promote initiatives directed at closing the 'gender data gap',*** fostering inclusive neuroscience research (e.g., Ann S. Bowers Women's Brain Health Initiative;[42] Women's Brain Project)
	• ***Women often serve as "Chief Medical Officers" for their families***, making health decisions, managing health costs, and providing informal care.[40]<br>• ***Women face higher risks of economic hardship*** creating a major barrier to gender equity.[40] In Europe, unpaid caregiving prevents 7·7 million women from working.[43] Resultant gender pension gaps (OECD average: 26%) [44] contribute to higher poverty levels in older women.	• ***Provision of effective carer support is essential:*** policies should ensure access to training, financial aid, pensions, and preventive healthcare.<br>• ***Fund scalable educational carer programs*** (e.g., WHO iSupport, for dementia caregivers) to effectively upskill carers and alleviate caregiver burden.[45]
Infrastructure	• ***Infrastructure design in urban and rural areas*** is pivotal to the inclusion of people with neurological conditions, their carers, and families, and in enabling brain healthy behaviours.<br>• ***Public space and transportation access barriers:*** a dearth of disability-inclusive infrastructure in many LMICs hinders societal participation of people with neurological conditions.	• ***NBHP can inform infrastructure policies (urban planning, housing, transport)***, drawing on frameworks like the *Lancet* "Series on urban design, transport, and health"[46] and the emerging discipline *neurourbanism.*[47]<br>• ***Policies should incentivise brain healthy educational/workplace environments*** (i.e., promoting safe, smoke-free, inclusive, *neurodiverse*, non-sedentary, and flexible structures).
	• ***Road and vehicle safety:*** LMICs are disproportionately affected by traumatic brain injury (TBI) and spinal cord injury from road traffic accidents.[48] Insufficient pre-hospital care structures and limited post-acute care access aggravate clinical outcomes. [48]	• ***NBHP should reinforce Member States' road safety commitments,*** i.e., WHO's Decade for Action on Road Safety plan, aiming to prevent at least 50% of traffic-related deaths and injuries by 2030.

Environment & climate change	• ***Negative environmental impacts*** on neurological/brain[49,50] and mental health[51] related to<br>○ ***pollution*** (i.e., air, noise, light)<br>○ ***climate change:*** *direct* (rising temperatures, natural disasters, neurotropic communicable disease) and *indirect* effects (sequelae of forced displacement, "eco-anxiety")<br>○ ***neurotoxic substance exposures*** (i.e., pesticides, nanoplastics, heavy metals)<br>• ***LMICs are disproportionately affected*** by "environmentally mediated brain disease" given limited environmental protection measures, infrastructure constraints, and poor health and safety regulations.[49]	• ***Align NBHP with environmental policies*** given bidirectional links between anthropogenic impacts and the *neural exposome* (= the totality of exposures impacting neurological health, including environmental toxins, psychosocial factors, diet, physical activity, sleep, and the microbiome).[52]<br>• ***Promote access to urban green and blue spaces (UGBS)***. UGBS positively impact mental/brain health[53] and can yield significant public health, social, economic, and environmental gains.[54]<br>• ***Brain health promotion may enhance eco-friendly behaviours*** and 'ecological intelligence' by strengthening metacognitive abilities, essential to belief revision and influencing environmental attitudes/behaviours.[55,56]
Food & agriculture	• ***Adequate nutrition*** (i.e., healthy, balanced diets; absence of under-/overnutrition) is vital for brain health across the life course- from promoting breastfeeding to preventing nutritional deficiencies in older age.[1,34]<br>• ***Adverse effects on brain health*** include agricultural (e.g., use of pesticides), sanitary (e.g., foodborne neurotropic infections like neurocysticercosis), and industrial factors (e.g., ultra-processed foods).	• ***Tackling the commercial determinants of health***,[57] e.g., through restricting marketing of unhealthy foods, taxation (sugar, salt, fat), incentivising product reformulation, food labelling and consumer education measures.[58]<br>• ***NBHP can inform public-facing national food and nutrition policies***, including those promoting breastfeeding, healthy food programmes in schools, knowledge building and consumer awareness on brain healthy foods.[1,59]
Technology & digitalisation	• ***Technological advancements:*** the convergence of generative AI and neurotechnology (brain-computer interfaces, neurorobotics) harbours vast therapeutic potential for neurology/brain health (e.g., brain-to-text decoding for speech neuroprostheses[60]).<br>• ***Effects of digitalisation on brain health*** are multifaceted,[61] impacting neurodevelopment, learning/memory, emotional intelligence and social behaviour. Risks for digital addiction, decision/ compassion fatigue, impaired attention, social isolation, and sleep disruption warrant further research.[61]	• ***Closing the 'knowledge translation gap':*** design policy structures promoting responsible integration of emerging AI applications into clinical neurology (cf. *Ethics & human rights*).<br>• ***WHO's regulatory considerations on artificial intelligence for health***[62] can guide global neurology stakeholders to facilitate safe and appropriate use of AI technologies/systems in clinical settings.<br>• ***NBHP should inform educational*** (i.e., digital literacy programs; responsible use of AI/ digital tools) ***and labour policies*** (i.e., addressing rapid workforce automation by re-/upskilling employees[15]).
Ethics & human rights	• ***The emerging concept of 'dignity neuroscience'*** identifies robust neural correlates for human rights (i.e., agency, autonomy, self-determination, etc.), with direct implications for brain health.[63]<br>• ***Unprecedented ethico-legal challenges*** arising from generative AI (e.g., rights to education, employment, privacy, data protection etc.)[64] and neurotechnologies (e.g., rights to mental privacy, cognitive liberty).[65]	• ***Explore dignity neuroscience*** as a cross-culturally applicable ethical framework to inform and promote human rights policies globally.[63]<br>• ***Utilise available global guidelines*** to craft governance frameworks for responsible AI (e.g., UNESCO guidelines)[64] and neurotechnology (e.g., OECD guidelines[66]) development and use.
	• ***Discriminatory legislation and multi-level stigma*** against persons with neurological conditions like epilepsy still exist many countries, curtailing human rights, and diminishing quality of life and appropriate care.[67]	• ***NBHP should reinforce Member States' commitments*** to the UN Convention on the Rights of Persons with Disabilities.<br>• ***In updating legislation to protect the rights of people with epilepsy*** (IGAP SO 5.2), Member States can refer to the WHO-OHCHR Mental health, human rights, and legislation: guidance and practice[68] resource.

Abbreviations: *AI= artificial intelligence; LMICs= low- and middle-income countries; NBHP= neurological and brain health plans; OECD= Organisation for Economic Co-operation and Development; OHCHR= Office of the United Nations High Commissioner for Human Rights; SO= strategic objective; UN= United Nations; UNESCO= United Nations Educational, Scientific and Cultural Organization; WHO= World Health Organization*

Supplementary Table 3. Examples of emerging IGAP regionalisation/domestication initiatives and related opportunities across different geographical regions.

Region	Type/actor	IGAP-directed activities and related opportunities
**African Region**	***WHO-led IGAP regionalisation***	• The 2004 Epilepsy in the African Region Report: Bridging the Gap is updated with the latest epidemiological data to produce a comprehensive ***"IGAP situational analysis"***:<br>○ Led by the WHO Regional Office for Africa and co-developed with key civil society stakeholders.<br>○ Identified regional priority neurological conditions include stroke, dementia, Parkinson's disease, headache disorders, and epilepsy.<br>○ Status quo on research, health systems, treatment, prevention, palliative care, rehabilitation, and reintegration is covered.<br>○ Actionable short-, mid-, and long-term policy recommendations for regional IGAP implementation are provided.

		• ***Preceding IGAP***, the WHO AFRO Framework for Defeating Meningitis by 2030 constitutes a successful regionalization example of the “WHO Global strategy to defeat meningitis by 2030”,[69] complementing the forthcoming IGAP situational analysis.
	IGAP-directed civil society activities	• ***IGAP SO 1* and 5*:*** the IBE Trendsetters Project (led by Mauritius, South Africa, Kenya, and Eswatini)[70] and IBE Advocate’s Toolkit for Reducing Epilepsy Stigma in Africa.<br>• ***IGAP SO 2*:*** the EAN-AFAN “Brain health across the lifespan” Regional Teaching Courses in Sub-Saharan Africa, the WNFO Global Neurology Consortium and Toolkits for Africa, WFNR community based rehabilitation project in LMICs, and the ICNA “Strategic plan to upskill capacity for multidisciplinary child neurology care in Zimbabwe” address the neurological treatment gap by building health professional capacity and resolving workforce and infrastructure shortages in the region. ICNA’s initiative may serve as a model project for expanding professional paediatric neurology care to other Sub-Saharan African countries where such care is virtually absent.[71]
**European Region**	***Opportunities for IGAP regionalisation***	• ***The European Commission (EC)*** and key European civil society organisations (EBC, EFNA, EAN, EPNS) create a distinctive policy environment, fostering synergistic development and implementation of quality NBHP across Europe.<br>o EC’s Healthier together – EU non-communicable diseases initiative is advancing IGAP directives by supporting countries with effective policies to reduce major NCD burdens:<br>▪ The initiative spans 2022-2027 with five priority areas, including a strand on “mental health and neurological disorders”.<br>▪ IGAP-relevant action areas for EU countries include mental health-in-all-policies, person-centred integrated care for neurological conditions, prevention and early detection (IGAP SO 3*), national plans (stroke; IGAP SO 1*), stigma reduction (mental health, dementia; IGAP SO 5*), and continuous EU-level monitoring and harmonised data registries to address existing health inequalities (IGAP SO 4*).<br>o The EU Brain Health Partnership aims to advance IGAP directives by accelerating the delivery of preventive, diagnostic, therapeutic and care solutions to foster brain health (including mental health):<br>▪ The initiative will span 2025-2027 within the second Horizon Europe Strategic Plan. EU funding will be matched by industry and governmental funds, pooling and mobilising resources towards common objectives.<br>▪ Framed within UN SDG 3.4, EC’s Healthier Together Initiative, and WHO’s Mental Health Action Plan and IGAP, expected impacts include research translation into innovative products for earlier, accurate diagnosis and more effective treatments (IGAP SO 2*), successful prevention and care strategies (IGAP SO 3*), and identifying best practices and implementable public health policies (IGAP SO 1*).<br>• ***The European Health Data Space*** (EHDS) is poised to advance IGAP directives as a health data governance framework:<br>o EHDS seeks to *1)* empower individuals through enhanced digital access to personal health data, *2)* foster a genuine single market for digital health services and products, and *3)* implement strict rules for using non-identifiable health data for research, innovation, policy-making and regulatory activities.<br>o Active neurology stakeholder involvement in EHDS development is crucial for scaling neurological research and innovation (IGAP SO 4*) and aiding policymakers in evidence-informed national NBHP development (IGAP SO 1*). Actions should encompass raising public awareness, promoting digital skills for trust/participation in data sharing, enhancing neurological infrastructure for better data collection, and advocating for regional legislative harmonization.<br>o If effectively executed, this regional model could offer valuable lessons, potentially serving as a blueprint for adaptation in other regions and/or the creation of a global health data governance framework.
	IGAP-directed civil society activities	• ***IGAP SO 1*:*** the EAN Brain Health Strategy[4] and Brain Health Mission support 47 national neurological societies to implement IGAP by providing advocacy training tools, materials and guidelines.<br>• ***IGAP SO 4*:*** to globally scale intersectoral brain research, the EBC, with FENS and IBRO, launched the Global Partnerships in Brain Research initiative. Stakeholders are united to resolve brain-related challenges “through knowledge and data sharing, capacity building, funding and policy.”
**The Americas**	***IGAP domestication in North America***	• ***United States Congress Members*** introduced a resolution designating September 15 as “National Brain Health Day”, in tandem with AAN’s inaugural 2022 Brain Health Summit (IGAP SO 1*).<br>• ***Civil society efforts:*** the AAN Brain Health Action Plan was launched in 2023 as a multi-year initiative addressing brain health across the lifespan, incorporating foetal, adolescent, adulthood, maturity, and senescence epochs (IGAP SO 1* and 3*).
	IGAP domestication in Latin America	• ***PAHO-led efforts:***<br>o Aligned with IGAP targets, regionalisation of the “WHO Global strategy to defeat meningitis by 2030”[69] will deliver a 2030 regional roadmap:<br>▪ Objective: to reduce meningitis cases by 50% and deaths by 70%.<br>▪ Priorities: large-scale awareness raising (targeting the public and decision-makers), fortifying key health system pillars (prevention, diagnosis, epidemiological surveillance, access to medical services, and care for patients with post-meningitis conditions).<br>o IGAP baseline mapping: efforts are underway to collate IGAP-relevant epidemiological, health services and systems data to identify regional priority neurological conditions and derive recommended policy actions.<br>• ***Civil society efforts*** encompass IGAP domestication projects and disease-specific regional initiatives:

		○ The first multi-stakeholder workshop on IGAP domestication (Santiago, Chile, 2023) convened by IBE, LICHE, and PAHO, involved people with epilepsy, families and carers, advocates, policymakers, healthcare professionals, and civil society organisations. This pilot project aimed to strengthen intersectoral alliances and establish consensus for IGAP implementation in Chile and the broader region (IGAP SO 1* and 5*).<br>○ A Latin American Brain Health Institute (BrainLat) was established as a regional hub devoted to research, capacity building, implementation science, and education. Additionally, the Alzheimer's Association and Global Brain Health Institute launched the Capacity Building in International Dementia Research (CBIDR) Program, a regional funding initiative building scientific investigation capacity in institutions within LMICs (IGAP SO 4*).
**South Asia**	***IGAP-directed civil society activities***	• ***A multi-stakeholder IGAP symposium*** "Epilepsy & Society in the Era of WHO-IGAP" (Jaipur, India, 2023), led by IBE, was held to foster intercultural exchange, cross-country collaboration, person-centred approaches, and partnerships for regional IGAP implementation (IGAP SO 1* and 5*).
	India-focussed IGAP activities	• ***The Government of India*** has introduced two flagship programs, TELEMANAS at national level and Karnataka Brain Initiative at state level, to improve accessibility and availability of neurological and mental health services in India (IGAP SO 2*).<br>• ***IGAP-directed civil society activities:***<br>○ Neurological stakeholders, including OneNeurology Ambassadors, sensitized national lawmakers at the Delhi State Legislative Assembly (October 10, 2022) about brain and mental health to elevate awareness and policy prioritisation (IGAP SO 1*).<br>○ For World Brain Day in India 2022, the IAN conducted a myriad of awareness initiatives across the country (IGAP SO 3*).<br>○ The ILAE is supporting IGAP implementation initiatives targeting the epilepsy treatment gap, including successful pilot projects on home-based care and provision of medications by primary-care personnel, enhancing seizure control and treatment adherence (IGAP SO 2* and 5*).<br>○ The INPCS international certificate course on neuropalliative care and train-the-trainer course aim at developing palliative care structures in LMICs, where 80% of palliative care needs exist (IGAP SO 2*).[72,73]

****IGAP Strategic Objectives:*** *SO 1 - Raise policy prioritization and strengthen governance; SO 2 - Provide effective, timely and responsive diagnosis, treatment, care; SO 3 - Implement strategies for promotion and prevention; SO 4 - Foster research and innovation and strengthen information systems; SO 5 - Strengthen the public health approach to epilepsy*

Abbreviations: *AFAN= African Academy of Neurology; EAN= European Academy of Neurology; EBC= European Brain Council; EC= European Commission; EFNA= European Federation of Neurological Associations; EPNS= European Paediatric Neurology Society; EU= European Union; FENS= Federation of European Neuroscience Societies; IAN= Indian Academy of Neurology; IBE= International Bureau for Epilepsy; IBRO= International Brain Research Organization; ICNA=International Child Neurology Association; IGAP= Intersectoral Global Action Plan on Epilepsy and Other Neurological Disorders 2022-31; ILAE= International League Against Epilepsy; INPCS= International Neuropalliative Care Society; LICHE= Liga Chilena Contra La Epilepsia; LMICs= low- and middle-income countries; PAHO= Pan American Health Organization; SO = strategic objective; WFNR= World Federation for Neurorehabilitation; WHO= World Health Organization; WNFO= World Neurology Foundation*

Supplementary Table 4. Examples of policy frameworks and agendas relevant to neurological/brain health and IGAP directives (list non-exhaustive).

**Type**	**Policy framework/agenda**
Health-related	WHO's Triple Billion targets
	Geneva Charter for Well-being
	WHO implementation roadmap for the WHO NCD-GAP 2023-2030
	Universal Health Coverage Action Agenda (UHC2030)
	WHO Model List of Essential Medicines
	WHO Rehabilitation 2030 Initiative and World Rehabilitation Alliance (WRA)
	WHO resolution on strengthening palliative care
	WHO defeating meningitis 2030 global roadmap[69]
	WHO Framework for Meaningful Engagement
Beyond health	UN 2030 Agenda for Sustainable Development
	UN Convention on the Rights of the Child
	UN Convention on the Rights of Persons with Disabilities
	The Paris Agreement
	UN Women Strategic Plan 2022–2025; Women's Brain Project
	OECD Neuroscience-inspired Policy Initiative
	UNESCO Recommendation on the Ethics of Artificial Intelligence
	OECD Council Recommendation on Responsible Innovation in Neurotechnology
	WHO-OHCHR Mental health, human rights, and legislation: guidance and practice[68]
	Neuroscience-inspired bioethical frameworks (e.g., Dignity Neuroscience[63], NeuroRights[74]

Abbreviations: NCD-GAP= WHO global action plan for the prevention and control of noncommunicable diseases; OECD= Organization for Economic Cooperation and Development; OHCHR= Office of the United Nations High Commissioner for Human Rights; UN= United Nations; WHO= World Health Organization

References

1 World Health Organization. Optimizing brain health across the life course: WHO position paper. Geneva, 2022 https://www.who.int/publications/i/item/9789240054561 (accessed Aug 16, 2022).
2 World Health Organization. Parkinson disease: a public health approach. Technical brief. Geneva, 2022.
3 World Health Organization. Improving the lives of people with epilepsy: a technical brief. Geneva, 2022.
4 Bassetti CLA, Endres M, Sander A, *et al.* The European Academy of Neurology Brain Health Strategy: One brain, one life, one approach. *Eur J Neurol* 2022; **29**: 2559–66.
5 Rost NS, Salinas J, Jordan JT, *et al.* The Brain Health Imperative in the 21st Century—A Call to Action. *Neurology* 2023; **101**: 570–9.
6 World Health Organization. Intention to action series: people power. Perspectives from individuals with lived experience of noncommunicable diseases, mental health conditions and neurological conditions. Geneva, 2023.
7 World Health Organization. Towards a dementia inclusive society: WHO toolkit for dementia-friendly initiatives (DFIs). Geneva, 2021.
8 World Health Organization. Risk reduction of cognitive decline and dementia: WHO guidelines. Geneva, 2019.
9 Dawson WD, Booi L, Pintado-Caipa M, *et al.* The Brain Health Diplomat's Toolkit: supporting brain health diplomacy leaders in Latin America and the Caribbean. *The Lancet Regional Health – Americas* 2023; **28**: 100627.
10 Wijeratne T, Bassetti CLA, Grisold W, *et al.* Brain health for all on World Brain Day 2022. *Lancet Neurol* 2022; **21**: 772–3.
11 The Lancet Global Health. Mental health matters. *Lancet Glob Health* 2020; **8**: e1352.
12 Owolabi MO, Thrift AG, Mahal A, *et al.* Primary stroke prevention worldwide: translating evidence into action. *Lancet Public Health* 2022; **7**: e74–85.
13 Wimo A, Seeher K, Cataldi R, *et al.* The worldwide costs of dementia in 2019. *Alzheimers Dement* 2023. DOI:10.1002/ALZ.12901.
14 Olesen J, Gustavsson A, Svensson M, Wittchen HU, Jönsson B. The economic cost of brain disorders in Europe. *Eur J Neurol* 2012; **19**: 155–62.
15 The Lancet. The future of work and health. *The Lancet* 2023; **402**: 1299.
16 Bertram MY, Sweeny K, Lauer JA, *et al.* Investing in non-communicable diseases: an estimation of the return on investment for prevention and treatment services. *The Lancet* 2018; **391**: 2071–8.
17 Feigin VL, Owolabi MO, Abanto C, *et al.* Pragmatic solutions to reduce the global burden of stroke: a World Stroke Organization–Lancet Neurology Commission. *Lancet Neurol* 2023; **0**. DOI:10.1016/S1474-4422(23)00277-6.
18 Lock SL. The benefits of brain health to our economies. *Nature Aging 2023 3:1* 2023; **3**: 1–2.
19 Ibáñez A, Legaz A, Ruiz-Adame M. Addressing the gaps between socioeconomic disparities and biological models of dementia. *Brain* 2023; **146**: 3561–4.
20 Scott AJ. The longevity economy. *Lancet Healthy Longev* 2021; **2**: e828–35.
21 Herre B. The world has recently become less democratic. 2022.
22 Economist Intelligence Unit. Democracy Index 2022: Frontline democracy and the battle for Ukraine. 2022.
23 Bollyky TJ, Templin T, Cohen M, Schoder D, Dieleman JL, Wigley S. The relationships between democratic experience, adult health, and cause-specific mortality in 170 countries between 1980 and 2016: an observational analysis. *The Lancet* 2019; **393**: 1628–40.
24 Winter SF, Angeler DG, Dawson WD, *et al.* BRAIN HEALTH-DIRECTED POLICYMAKING: A NEW CONCEPT TO STRENGTHEN DEMOCRACY. Washington D.C. : Brookings Institution, 2022.
25 Weinbaum C, Khan O, Thomas TD, Stein BD. Neurodiversity and National Security: How to Tackle National Security Challenges with a Wider Range of Cognitive Talents. 2023. DOI:10.7249/RRA1875-1.
26 Gore KL, Cherney S, Shih RA, Girven RS. Could Dementia in the National Security Workforce Create a Security Threat? Santa Monica, CA: RAND Corporation, 2023 DOI:10.7249/PEA1779-1.
27 Jost JT, Nam HH, Amodio DM, Van Bavel JJ. Political Neuroscience: The Beginning of a Beautiful Friendship. *Polit Psychol* 2014; **35**: 3–42.
28 Zmigrod L, Tsakiris M. Computational and neurocognitive approaches to the political brain: key insights and future avenues for political neuroscience. *Philos Trans R Soc Lond B Biol Sci* 2021; **376**. DOI:10.1098/RSTB.2020.0130.
29 Kubzansky LD, Epel ES, Davidson RJ. Prosociality should be a public health priority. *Nature Human Behaviour 2023* 2023; : 1–3.

30 Beddington J, Cooper CL, Field J, *et al.* The mental wealth of nations. *Nature 2008 455:7216* 2008; **455**: 1057–60.
31 Occhipinti J-A, Buchanan J, Hynes W, *et al.* Estimating the Mental Wealth of nations: valuing social production and investment. *Nature Mental Health 2023 1:4* 2023; **1**: 247–53.
32 Smith E, Ali D, Wilkerson B, *et al.* A Brain Capital Grand Strategy: toward economic reimagination. *Mol Psychiatry* 2021; **26**: 3–22.
33 Matyas N, Keser Aschenberger F, Wagner G, *et al.* Continuing education for the prevention of mild cognitive impairment and Alzheimer's-type dementia: a systematic review and overview of systematic reviews. *BMJ Open* 2019; **9**. DOI:10.1136/BMJOPEN-2018-027719.
34 Cusick SE, Georgieff MK. The Role of Nutrition in Brain Development: The Golden Opportunity of the "First 1000 Days". *Journal of Pediatrics* 2016; **175**: 16–21.
35 Walker SP, Wachs TD, Meeks Gardner J, *et al.* Child development: risk factors for adverse outcomes in developing countries. *Lancet* 2007; **369**: 145–57.
36 Black MM, Walker SP, Fernald LCH, *et al.* Early childhood development coming of age: science through the life course. *The Lancet* 2017; **389**: 77–90.
37 World Health Organization. Global Scales for Early Development (GSED) v1.0. Geneva, 2023.
38 Thompson BL, Levitt P, Stanwood GD. Prenatal exposure to drugs: effects on brain development and implications for policy and education. *Nat Rev Neurosci* 2009; **10**: 303.
39 Feigin VL, Vos T, Nichols E, *et al.* The global burden of neurological disorders: translating evidence into policy. *Lancet Neurol* 2020; **19**: 255–65.
40 Economist Impact. Sex, gender and the brain: towards an inclusive research agenda. 2023.
41 The Lancet Neurology. Sex, gender, and the cost of neurological disorders. *Lancet Neurol* 2023; **22**: 367.
42 Jacobs EG. Only 0.5% of neuroscience studies look at women's health. Here's how to change that. *Nature* 2023; **623**: 667–667.
43 European Commission. Factsheet: Care Strategy. Brussels, 2022.
44 OECD. Pensions at a Glance 2021: OECD and G20 Indicators. Paris, 2021.
45 Seeher K, Cataldi R, Chowdhary N, Kolappa K, Dua T. The need for a better global dementia response. *Lancet Neurol* 2022; **21**: 115.
46 Giles-Corti B, Moudon AV, Lowe M, *et al.* What next? Expanding our view of city planning and global health, and implementing and monitoring evidence-informed policy. *Lancet Glob Health* 2022; **10**: e919–26.
47 Adli M, Berger M, Brakemeier EL, *et al.* Neurourbanism: towards a new discipline. *Lancet Psychiatry* 2017; **4**: 183–5.
48 Maas AIR, Menon DK, Manley GT, *et al.* Traumatic brain injury: progress and challenges in prevention, clinical care, and research. *Lancet Neurol* 2022; **21**: 1004–60.
49 Tshala-Katumbay D, Mwanza JC, Rohlman DS, Maestre G, Oria RB. A global perspective on the influence of environmental exposures on the nervous system. *Nature* 2015; **527**: S187–92.
50 Louis S, Carlson AK, Suresh A, *et al.* Impacts of Climate Change and Air Pollution on Neurologic Health, Disease, and Practice. *Neurology* 2023; **100**: 474–83.
51 Cianconi P, Betrò S, Janiri L. The Impact of Climate Change on Mental Health: A Systematic Descriptive Review. *Front Psychiatry* 2020; **11**: 74.
52 Tamiz AP, Koroshetz WJ, Dhruv NT, Jett DA. A focus on the neural exposome. *Neuron* 2022; **110**: 1286–9.
53 White MP, Elliott LR, Grellier J, *et al.* Associations between green/blue spaces and mental health across 18 countries. *Scientific Reports 2021 11:1* 2021; **11**: 1–12.
54 Hunter RF, Nieuwenhuijsen M, Fabian C, *et al.* Advancing urban green and blue space contributions to public health. *Lancet Public Health* 2023; **8**: e735–42.
55 De Beukelaer S, Vehar N, Rollwage M, Fleming SM, Tsakiris M. Changing minds about climate change: a pervasive role for domain-general metacognition. *Humanities and Social Sciences Communications 2023 10:1* 2023; **10**: 1–10.
56 Leeuwis N, van Bommel T, Alimardani M. A framework for application of consumer neuroscience in pro-environmental behavior change interventions. *Front Hum Neurosci* 2022; **16**. DOI:10.3389/FNHUM.2022.886600.
57 Gilmore AB, Fabbri A, Baum F, *et al.* Defining and conceptualising the commercial determinants of health. *The Lancet* 2023; **401**: 1194–213.
58 Kalra S, Verma M, Kapoor N. Commercial determinants of health: A critical component of the obesogenic environment. *Clin Epidemiol Glob Health* 2023; **23**: 101367.
59 Adan RAH, Cirulli F, Dye L, *et al.* Towards new nutritional policies for brain health: A research perspective on future actions. *Brain Behav Immun* 2022; **105**: 201–3.

60 Ramsey NF, Crone NE. Brain implants that enable speech pass performance milestones. *Nature 2023 620:7976* 2023; **620**: 954–5.
61 Small GW, Lee J, Kaufman A, *et al.* Brain health consequences of digital technology use. *Dialogues Clin Neurosci* 2020; **22**: 179.
62 World Health Organization. Regulatory considerations on artificial intelligence for health. Geneva, 2023.
63 White TL, Gonsalves MA. Dignity neuroscience: universal rights are rooted in human brain science. *Ann N Y Acad Sci* 2021; **1505**: 40–54.
64 UNESCO. Recommendation on the Ethics of Artificial Intelligence. Paris, 2022.
65 Drew L. Mind-reading machines are coming - how can we keep them in check? *Nature* 2023; **620**: 18–9.
66 OECD. Recommendation of the Council on Responsible Innovation in Neurotechnology, OECD/LEGAL/0457. 2019 https://legalinstruments.oecd.org/en/instruments/OECD-LEGAL-0457.
67 The Lancet Neurology. Time to end the stigma of epilepsy. *Lancet Neurol* 2023; **22**: 283.
68 World Health Organization and the United Nations (represented by the Office of the United Nations High Commissioner for Human Rights). Mental health, human rights, and legislation: guidance and practice. Geneva, 2023.
69 World Health Organization. Defeating meningitis by 2030: a global road map. Geneva, 2021.
70 Winter SF, Walsh D, Amos A, Secco M, Sofia F, Baker GA. The WHO intersectoral global action plan and epilepsy cascade target: Towards a roadmap for implementation. *Seizure* 2022; **103**: 148–50.
71 Wilmshurst JM, Catsman-Berrevoets C, Gilbert DL, *et al.* Access to Pediatric Neurology training and services worldwide: A survey by the International Child Neurology Association. *Neurology* 2023; : 10.1212/WNL.0000000000207633.
72 Lau C, Meaney C, Morgan M, Cook R, Zimmermann C, Wentlandt K. Disparities in access to palliative care facilities for patients with and without cancer: A retrospective review. *Palliat Med* 2021; **35**: 1191–201.
73 Baruah U, Sharma P, Thomas PT, Dhamija RK. Neuropalliative care in India - Barriers, challenges and future directions. *Ann Indian Acad Neurol* 2023; **26**: 107–11.
74 Baselga-Garriga C, Rodriguez P, Yuste R. Neuro Rights: A Human Rights Solution to Ethical Issues of Neurotechnologies. 2022; : 157–61.
